# Promoting radical healing to facilitate community capacity building among formerly incarcerated Black and Latino men with substance use disorders

**DOI:** 10.1002/ajcp.12816

**Published:** 2025-05-19

**Authors:** Richmond E. Hayes, Heather A. Jones, Ellen Benoit, Dora N. Watkins, Liliane C. Windsor

**Affiliations:** ^1^ Department of Educational Psychology University of Illinois at Urbana‐Champaign Champaign Illinois USA; ^2^ School of Social Work University of Illinois at Urbana‐Champaign Champaign Illinois USA; ^3^ Mandel School of Applied Social Sciences Case Western Reserve University Cleveland Ohio USA

**Keywords:** capacity building, clinical intervention, critical consciousness, formerly incarcerated, radical healing, substance use

## Abstract

This study reports on a qualitative thematic analysis of secondary data from group session recordings collected as part of the *Community Wise* Optimization Trial. *Community Wise* is a multilevel behavioral intervention designed to increase critical consciousness and reduce substance use among formerly incarcerated men living in predominantly Black and historically disinvested communities (BHDC). Radical healing is a process of recovering from the trauma of oppression based on identification with historically marginalized groups. The current analysis sought to examine if there is evidence of radical healing components (critical consciousness, radical hope, strength and resistance, cultural authenticity and self‐knowledge, and collectivism or emotional and social support) in *Community Wise's* group sessions. Results revealed evidence of all radical healing components in *Community Wise* participants' rich narratives as they engaged in critical dialogue and explored ways to improve their communities. Given a lack of culturally relevant approaches, this study's findings provide supporting evidence for the incorporation of the radical healing framework in interventions seeking to enhance treatment outcomes and address social determinants of health in BHDC.

## Highlights

- Critical consciousness is the first step and beginning of the radical healing process.
- Participants' experiences of the intervention can be understood as a journey toward radical healing.
- The radical healing framework's components were engaged unconsciously throughout the intervention.
- Interventions should adopt the psychological framework of radical healing in predominately BHDC.

## INTRODUCTION

Predominantly Black and historically disinvested communities (BHDC) are defined as communities that include disproportionately higher rates of Black residents and who have been historically and systematically excluded from social and economic developments in the United States because of structural racism. Structural racism is the complex system of policies, practices, cultural representations, and other norms that work together to perpetuate health inequities between Whites and People of Color (Braveman et al., 2022). For instance, between 1935 and 1940, the Home Owners' Loan Corporation graded the “residential security” of thousands of American neighborhoods by determining investment risks for investors (Greer, 2013, p. 275). Neighborhoods with Black populations were automatically excluded from financial investment. As a result, these communities still struggle with access to high‐quality education, meaningful employment, safe and affordable housing, and quality health care services (Swope et al., 2022). Today, BHDC are comprised of residents who earn less than $20,000 annually and also include a significant number of individuals who identify with Latino groups (Spader et al., 2017).

Given the impact of structural racism on BHDC, it is not surprising that Black Americans constitute 14% of the U.S. population but 33% of the state and federal prison population, driven by racist law‐and‐order strategies such as racial profiling (Alexander, 2010). Simultaneously, Hispanic or Latino Americans constitute 20% of the U.S. population and 23% of those incarcerated in state and federal prisons (U.S. Census Bureau, 2022). When released from incarceration, Black and Latino Americans often return to BHDC (Benzow et al., 2020). It is critical, therefore, to address the disproportionate impacts of structural racism on BHDC through multilevel interventions that go beyond a focus on individual behavior and move away from character judgment.

Group interventions with historically disinvested and racialized communities that encourage the radical belief that change is possible amidst oppression can improve intervention outcomes. Participants can reflect on the human condition and evaluate the amounts of freedom they themselves—as well as the collective—have to change their oppressive and unequal social circumstances. Ultimately, a shift can occur, away from isolation and a lack of concern for their community and towards collective action and radical healing.

The practice of moving toward freedom through critical reflection is influenced by Brazilian educator Paulo Freire's work with adults who were unable to read or write (Freire, 1970). He argued dominant elites use the “banking” concept of education to “fill” the minds of the oppressed “with slogans which create even more fear of freedom” (Freire, 1970, p. 84). Liberation requires critical consciousness, which can be developed through an approach to education where learning is horizontal rather than vertical (i.e., “teacher‐student” and “students‐teachers”) and where oppressive messages are presented as critical areas for exploration (Freire, 1970, p. 99). It also demands that dialogue—organized around existential questions and prospective interests—foster critical reflection, invite responses, and provoke movement. Freire (1970) presented sketches or photographs to participants for “decoding,” in a process characterized by a shift from abstract representations to the concrete reality and challenges they must overcome (pp. 96–97). Simply put, through critical reflection and dialogue, individuals collectively identify obstacles on the road to liberation and devise strategies of resistance.

Since developing critical consciousness is a collective endeavor, the process encourages social support and solidarity. In a review of research on how Black people cope with racism, Jacob et al. (2023) found that both Black men and Black women most frequently reported using social support from family, friends, and the community as their primary coping strategy. Along with religion and spirituality (e.g., attending church and praying), other commonly used coping strategies included engaging in avoidance activities (i.e., disregarding or minimizing race‐related stressors) as well as activating problem‐focused coping strategies (i.e., actively handling race‐related stressors). In their investigation, Black women were more likely to confront stressful, race‐related situations and to speak out, whereas Black men were more likely to attempt to avoid race‐related stress, a difference the authors of the article speculate may be attributable to the ways in which Black men in Western societies are punished for exercising their agency. Noting the risks and benefits associated with not responding to and with confronting racism, the authors recommend direct strategies like activism, which are associated with more beneficial outcomes when they are utilized at the community rather than the individual level.

The idea that individual resistance can lead to negative health effects in members of marginalized populations is a factor in Goodkind and colleagues' (2020) study of an empowerment program to raise critical consciousness among groups of Black adolescent girls. Citing research demonstrating resilience is often measured in terms defined by White middle‐class male standards, the authors argue oppressed individuals—such as Black adolescent girls who challenge injustice to defend their well‐being—are frequently labeled as deviant and subjected to disciplinary consequences. To encourage Black girls to change oppressive systems rather than adapt to them, the empowerment program was designed to promote collective resilience, grounded in critical consciousness and a positive group identity. Post‐intervention survey and qualitative results indicated increased social bonds and collective action and included findings that participants were more likely to perceive injustice and less likely to internalize racial and gender stereotypes. The effects of the group sessions demonstrate the possible utility of interventions that challenge individual treatment models, which place all the responsibility of change on individuals and ignore the impact of structural racism.

## FRAMEWORK OF RADICAL HEALING

One promising framework for use in multilevel health interventions is radical healing—a process of recovering from the trauma of oppression based on identification with certain classes, races, genders, and/or sexual orientations. Radical healing may be the right framework for multilevel health interventions, given its offering of healing historical trauma and empowerment for People of Color, which address oppression and promote health equity (Amaro et al., 2021). As a psychological framework, radical healing is grounded in the psychology of liberation, Black psychology, ethnopolitical psychology, and intersectionality. It posits that racial and social justice are necessary conditions for healing (French et al., 2020). Radical healing is a collective process with five components: critical consciousness; radical hope; strength and resistance; cultural authenticity and self‐knowledge; and collectivism or emotional and social support (French et al., 2020).

*Critical consciousness* is seen as the first step, beginning at the individual level with critical reflection, which involves understanding oppression and rejecting inequity. As described above, it fosters political efficacy, or confidence in one's ability to facilitate change, which incites critical action (Freire, 1970).

*Radical hope* incorporates political efficacy and critical action, with the belief that acts of resistance will produce desired results. High levels of hope have commonly been affiliated with positive results (e.g., well‐being, quality of life, life satisfaction, improved problem‐solving abilities, improved coping strategies, and better treatment outcomes in psychotherapy), and having personal goals is a specific element of hope associated with well‐being (Constantino et al., 2018; Johnson et al., 2020; Marques & Gallagher, 2017). When Stearns et al. (2018) explored hope among incarcerated women, they found their goals included “jobs, sobriety, education, volunteerism, [and] family reunification” (p. 404). In the hope literature, messages of hope can be found in stories of others' experiences, such as television programming featuring Black fathers teaching life lessons, whose stories reflect hope and stand in stark contrast to the negative images of Black men that dominate media (Boddie et al., 2024).

*Strength* is required for *resistance* to oppression and refers to communities working together to dismantle barriers to their collective well‐being. “Everyday resistance” is a strategy that people who are oppressed employ in situations where overt, collective action is not safe or feasible and that classifies steps such as those taken to maintain one's overall well‐being as acts of resistance (Rosales & Langhout, 2020).

*Cultural authenticity* involves *self‐knowledge* and ultimately rejects European ideologies as absolute truths and reestablishes pride in one's ethnic heritage and ancestral knowledge (French et al., 2020). Becoming aware of discrimination as a systemic issue in addition to being a personal one is found in the process of developing critical consciousness to resist anti‐Black racism (Goodkind et al., 2020; Mosley et al., 2021). Research with formerly incarcerated young Black and Latino men has found that ethnic pride might be a source of strength in successful community re‐entry, given its association with less involvement in illegal activities and with positive attitudes toward avoiding violence in conflict situations (Upadhyayula et al., 2017). Other research, with formerly incarcerated young Black men in alternative schools, has found that culturally relevant curricula and the ability to self‐direct learning can enhance engagement with education (Lea et al., 2020).

The final component of radical healing is *emotional and social support*. It establishes connections with one's community informed by shared racial and ethnic cultural values, thus reinforcing solidarity and *collectivism* (French et al., 2020). As discussed above, social support is frequently utilized by Black adults as a strategy to cope with racism (Jacob et al., 2023). In a study of racism‐based traumatic stress among Black and Latino young adults, participants' definitions of collective care highlighted community organizing, activism, and the importance of affinity/cultural space (Gomez et al., 2023).

The five components of radical healing operate together in the context of movement from environments of oppression and hate to spaces of justice and liberation. Radical healing is a multisystemic framework that integrates several existing theories to create a comprehensive, multi‐level, and ecological approach to health and wellness. Empirical studies, utilizing this comprehensive model of how people of color can heal from oppression, provide insights into how its components can promote well‐being and liberation across various contexts and populations.

Notable applications of the radical healing framework include a qualitative metasynthesis of 106 studies examining the lived experiences of Black queer people through lenses made from the radical healing and psychopolitical well‐being frameworks (Mosley et al., 2021). Among the findings of the metasynthesis was the emergence of four major themes: the omnipresence of oppression, the absence of safety, the pain and possibilities in the cycle of HIV stigma, as well as self‐acceptance and social support. The study emphasized a pervasive lack of physical and emotional safety; identified self‐acceptance and social support as vital components of personal liberation; and demonstrated how cultural authenticity and resilience portrayed through storytelling can be a key strategy for enhancing knowledge and fostering connection. Overall, the findings illustrate how radical hope and collectivism facilitate healing.

Another study investigated the effects of racial discrimination and COVID‐19‐related stress on suicide risk among Black emerging adults, using the radical healing framework to understand moderating factors (Brooks Stephens et al., 2024). The main finding was that critical consciousness, resilience, and cultural authenticity significantly reduced suicide risk. Moreover, it found that participants who exhibited higher levels of racial pride and sociopolitical awareness were better equipped to navigate systemic stressors—which underscores the importance of these concepts in fostering psychological well‐being.

Besides that, a qualitative study by Leath et al. (2024) used the radical healing framework to analyze how Black mothers raise “free Black children.” Three themes emerged from their exploration: promoting pro‐Black critical consciousness, encouraging self‐authenticity through socio‐emotional support, and building strength and resistance through community care. Mothers emphasized the importance of equipping their children with the tools to understand and resist systemic oppression, while fostering their emotional well‐being and social connections, showcasing how concepts like critical consciousness and collectivism are embedded in parenting practices.

Despite these studies demonstrating the applicability of the radical healing framework, several gaps remain. One gap is the ongoing need to explore and provide evidence to support the arguments of the framework. What is proposed is a multisystemic psychological framework of radical healing for communities of color, and considering that the radical healing framework is still relatively new, the experience of intersectional identities among People of Color (e.g., formerly incarcerated Black and Latino men) as well as how collective liberation is fostered remain critical areas for future inquiry. Another gap involves the need for more culturally relevant and collectivistic approaches to capture the lived experiences of communities of color. To understand the interlocking systems of oppression, the authors of the framework of radical healing discourage a narrow utilization of research methodologies. Instead, they encourage the use of multiple research methodologies to provide a broader and more inclusive perspective.

On top of these gaps, there continues to be a need to capture the collectivistic nature of resilience in communities of color. Empirical studies have begun to demonstrate the utility of the radical healing framework in addressing the lived experiences of People of Color, and these applications highlight the importance of critical consciousness, resilience, cultural authenticity, and collectivism in promoting well‐being and liberation. However, contributions focusing on collective liberation among other vulnerable groups, as well as addressing calls for methodological diversity, are still necessary. Addressing these gaps will not only enhance the psychological framework of radical healing's applicability but also provide deeper insights into the multifaceted experiences of communities of color navigating systemic oppression.

## COMMUNITY WISE OPTIMIZATION TRIAL: THE PARENT STUDY

Data for the current study come from a randomized clinical trial (RCT) to optimize *Community Wise*, an intervention designed to address factors contributing to substance use at the individual, social, and community levels (Windsor et al., 2024). Grounded in critical consciousness theory, *Community Wise* conceptualizes substance use as a consequence of internalized and structural oppressions including racism, classism, and other forms of discrimination. The intervention was delivered to 602 formerly incarcerated men with histories of substance use disorder in weekly group sessions guided by either a peer or licensed facilitator. It included a Core component (Critical Thinking) and a combination of three other components—Critical Dialogue (CD), Capacity Building Project (CBP), and Quality‐of‐Life Wheel. Through facilitated group discussions participants were expected to develop critical consciousness by engaging in critical reflection and planning critical action to improve their own health and that of their communities. The testing of all possible combinations of components and of peer versus licensed facilitators yielded 48 groups. The RCT then implemented a randomized 2^4^ full factorial experimental design to identify what components or combination of components would be most effective in reducing substance use frequency and that could be delivered for less than $250 per person. Substance use was tracked monthly over 5 months through urinalysis and self‐report (Windsor et al., 2024). Findings from the RCT showed that Critical Thinking delivered by a peer facilitator alongside the CD and CBP components produced clinically and significantly greater reductions in substance use (Windsor et al., 2024).

## CURRENT STUDY

The current study is a qualitative, thematic analysis of group session transcripts of recordings collected during the *Community Wise* Optimization Trial. The current analysis sought to examine whether *Community Wise* promoted radical healing among its participants. More specifically, the study answers the following research questions: 1) Is there evidence of radical healing components in *Community Wise's* group session transcripts? 2) If so, how do participants discuss these components in their own words? Although radical healing was not explicitly a part of *Community Wise's* original design, critical consciousness (i.e., the first step of radical healing) was infused throughout the intervention.

## METHOD

The parent study was approved by the Institutional Review Board at the University of Illinois, Urbana Champaign (NIH R01MD010629; MPI Windsor & Benoit). All participants provided written informed consent and chose code names before attending any sessions. All sessions were audio‐recorded for clinical supervision and for data analysis. For this study, we analyzed data only from group sessions that delivered the optimized version of *Community Wise* (including Core Critical Thinking, CD, and CBP delivered by a peer facilitator) to examine if and how participants engaged in a radical healing process from intersecting forms of oppression (e.g., racism and classism, gendered racial and substance use discrimination). Below, we provide more details about each intervention component in the optimized version of *Community Wise*.

### Intervention component: Critical thinking

In the first two sessions of *Community Wise*, participants and the facilitator get to know each other. Together, they explore foundational concepts such as oppression, privilege, and the production of knowledge and beliefs. There is an emphasis on anti‐oppressive reflection, which encourages participants to question the information they have received as well as the ideologies they have accepted. Participants are guided by a handbook that includes an introduction to critical consciousness, a glossary, and text for each session that covers topic descriptions and assigned readings on health and inequities. The handbook was written at an eighth‐grade reading level, verified by Readable. The reading assignments were summaries of published articles, written at the same level.

During this component, prompted by reading assignments, participants discuss the use of science and research and reflect on each other's experiences and perspectives. For example, in Session 2, participants are presented with a universal theory/belief (e.g., the earth is round). Then, they are asked to prove this understanding/belief/knowledge and to provide evidence against the understanding. Ultimately, problems with not questioning assumed truths and with overlooking gaps in knowledge are discussed. The overall goal of this component is to examine materials about critical thinking and learn how to evaluate one's own way of thinking.

### Intervention component: Critical dialogue (CD)

The CD component prompts participants to think critically about specific forms of oppression over six sessions (i.e., Sessions 3, 5, 7, 9, 11, and 13). In particular, participants conduct a detailed analysis of unequal distributions of power, internalized oppression, and structural barriers to health and social equity. For example, they carefully examine the concepts of homophobia and sexism in addition to historical trauma and the long‐term impacts of slavery (Pinto et al., 2024). Each session in this component begins with participants interpreting an illustration, then sharing and comparing perspectives and experiences with other participants (Jemal et al., 2022).

For instance, in session 11, the facilitator shows participants an illustration of a happy, traditional White middle‐class family in front of their suburban home with their dog and then poses critical questions. They include queries such as “How does this family compare with the families in your neighborhood,” “What values do you think the parents in the illustration pass down to their children,” and “What values, behaviors, and expectations would you like to pass down to your family.” Following this, the facilitator reads a take‐home message which communicates that historical trauma is transmitted inter‐generationally through family relationships; that control of images and unreachable standards are powerful tools in the maintenance of the status quo; and that people can combat oppression and privilege by redefining family, family norms, and setting more realistic expectations. The goal of this component is to use critical questions to challenge deeply held beliefs, assess community challenges, and develop action to address concerns.

### Intervention component: Capacity building project (CBP)

The CBP component requires participants to apply critical reflection and critical action to issues of concern beyond their intervention group. Participants were tasked with applying their critical reflection skills in considering the conditions of their communities. They investigated the causes of those conditions and potential resources to aid in restoration in their communities. Over the course of the six CBP sessions (i.e., Sessions 4, 6, 8, 10, 12, and 14), participants developed a plan for feasible critical action to address specific challenges including homelessness, food insecurity/unmet physical health needs, and a lack of access to/sharing of community resources. Additionally, participants highlighted the need for youth afterschool programming/interventions, services for elderly community members, and community unity.

The development of CBP facilitated acts of resistance to the inequities in their communities. Acts of resistance were understood as any opposition to systems of dominance and oppression through self‐determination, agency, and collective action to confront such systems (Page & Woodland, 2023). Specific examples of acts of resistance implemented within the context of the CBP included one group drafting a letter to the mayor from the community addressing the issue of homelessness, as well as another group creating and distributing flyers to educate community members on their access to much‐needed resources. The overarching goal of this component is to promote agency and empower participants to take anti‐oppressive actions toward multisystemic changes that may strengthen their communities, reduce inequities, and reduce individual and community‐level harm.

### Researcher positionality

The authors of this article represent a diversity in experience and perspective as well as a diversity in both social and professional identities. Their social identities include various racial and ethnic backgrounds (e.g., Black/African American, White/Caucasian, and white Latina); genders (e.g., male and female); and social classes (e.g., working, middle, and upper‐middle class). All of the authors identify as researchers, in addition to multiple other professional identities—including psychologist, licensed clinical social worker/social worker, sociologist, artist, and activist. What is more, they have clinical experiences ranging from serving individuals incarcerated in federal prisons to working with formerly incarcerated Black men in community settings, on top of research experiences with marginalized populations using qualitative and mixed‐methods in culturally relevant intervention studies that focus on policy reform, structural/mental health inequities, healing justice, substance use/addiction, and the application of critical consciousness theory. Taken together, the authors are positioned to offer valuable insights, such as the lived experience perspective of a clinician, family member, and/or researcher who has been impacted by the criminal legal system.

### Participants

A total of 38 participants received the optimized intervention. Of this subsample, 94.7% identified as Black or African American and 5.3% as Hispanic or Latino (see Table 1). Age ranged from 22 to 67 (avg. 42.4). The subsample's religious affiliation and other demographics also resembled the overall RCT sample—with substance use, unstable housing, and unemployment being challenges for most participants (Windsor et al., 2024). The peer facilitator—who was 52 years old, employed full time, and had a history of substance use—identified as a married, formerly incarcerated African American Christian male from the same community as most of the participants.

**Table 1:** Sociodemographic characteristics for participants who received the optimized version of community wise.

Sample characteristics	*N*	(%)
Racial/ethnic		
Black/African American	36	94.7
Hispanic, Latino	2	5.3
Marital status		
Never married	27	71
Separated	3	7.9
Divorced	2	5.3
Married	4	10.5
Widowed	2	5.3
Employed		
Employed (35+ hours full time)	5	13.2
Employed (part time)	4	10.5
Unemployed (looking for work)	19	50
Unemployed (disabled)	6	15.8
Unemployed (not looking for work)	3	7.9
Other	1	2.6
Religion		
Christian	21	55.3
Muslim/Islam	8	21.05
Jewish	1	2.6
None of these	8	21.05
Housing		
Alone	3	7.9
With others	19	50
Institution (hospital, jail, prison, etc.)	0	0
Structured living	1	2.6
Homeless shelter	9	23.7
Homeless (in an abandoned building, in a car, on the street)	7	18.4

### Analysis

Three members of the research team conducted a thematic analysis (TA) on transcripts of the voice‐recorded group sessions of *Community Wise* participants who were randomized to receive the optimized intervention. The main analytical goal was to identify if the transcripts included evidence of the five radical healing components and, if so, how participants' narratives indicate a journey toward radical healing. The three analysts used template analysis, a codebook approach to TA, to analyze the data (Braun & Clarke, 2022; King & Brooks, 2017). First, they familiarized themselves with the entire data set, coding the data preliminarily and clustering the themes. Then, an initial template/coding framework was produced, applied, and developed utilizing a priori codes/themes (i.e., the components of radical healing). Additionally, although the analysts were open to modifications to the themes when developing the final template, the components proved meaningfully applicable throughout the analysis. Subsequently, after hierarchical coding, the analysts decided to prioritize the a priori themes, as they best represented the final interpretation of the data and were central to addressing the research questions in this write up.

Practically following template analysis procedures, this study's analysis began with preliminary coding, in which the three analysts listened to session recordings and transcribed relevant sections clustered around the radical healing components (i.e., critical consciousness, cultural authenticity and self‐knowledge, radical hope, strength and resistance, emotional and social support). Next, searching for content that addressed each element of radical healing, the analysts applied and further developed the initial template. Explicitly, the components informed the labels for the thematic evidence found in the qualitative data segments, and the radical healing framework served as a decision‐making tool to systematically code the qualitative data. For example, a participant's comment that might be described as cynical or hopeless may have been coded within the theme of radical hope, because radical healing involves movement from interlocking systems of oppression and hate toward a place of resistance that envisions justice and liberation (French et al., 2020).

Given the analysts' deep understanding of the elements of the “common collective struggle,” the meaning of each code was shaped by both the data content and the perceived locations of the participants in the overlapping spheres of feelings of oppression and visions of freedom (Ginwright, 2010, p. 63). In collaboration, they discussed the codes—not to reconcile coding differences, verify, or check for accuracy of the codes but “to enhance understanding, interpretation and reflexivity” (Braun & Clarke, 2022, p. 9). The analysts elicited each other's reactions to codes, challenged each other's thinking regarding codes, and made each other's assumptions explicit, which aided in their ability to make sense of the data. Finally, each of them independently read the transcripts and collaboratively created a matrix of codes and cases in Excel—using the final version of the template to develop a final interpretation. This article presents the findings of the template analysis, including illustrative quotes, structured around the radical healing components.

## FINDINGS AND DISCUSSION

Secondary thematic analysis of qualitative data revealed evidence of the presence of all radical healing components in *Community Wise* participants' rich narratives as they engaged in critical dialogue and explored ways to improve their communities. Analysis revealed that radical healing is not a linear process, but rather a journey. Although participants were exposed to the same material, as a part of the group endeavor, individuals could pursue different paths in the process of healing. As a result of the nature of radical healing, the radical healing components appeared without order, varying across participants and sessions. Below, we synthesize the evidence that emerged from each component of the radical healing framework with discussions of how the evidence compares to the extant literature.

### Critical consciousness

In all *Community Wise* group sessions, participants discussed oppression and privilege, including its impact on people and society (e.g., racism, classism, sexism) and their ability and willingness to engage in action to fight it (i.e., critical consciousness). Much of the intervention focused on promoting participants' interrogation of their own assumptions regarding the structure of power and sources of oppression, development of multiple interpretations of social realities, and generation of potential solutions. For instance, during the critical thinking session, participants read an article about research that debunked flawed, racist, and classist research about crack's impact on fetuses that was perpetuated by the media and influenced tough‐on‐crime policies during the 1980s (Zerai & Banks, 2002). One participant summarized how the reading changed his understanding about *crack babies* and connected it with his lived experience of betrayal from society and figures of authority:

They [policies, media, health care system] made a big deal over crack babies, but more babies were messed up by alcohol…I never did the research, so I didn't know. They got us thinking stuff is true that isn't true. It's what they show us, what we see…There's always something else there that they ain't telling us. That's why we need critical thinking (Session 2).

Although accurate information about how the use of different substances impacts fetuses in the womb was new to the participants, the idea that Black people were being harmed by false and racist information from those with power was not. In one group's first session, one participant asked his fellow group members if they thought there were more Black men going to college or going to prison.

Participant 2 (answers): Prison.

Participant 1: That's not true. That's what they want you to believe. I believed that, too, until I did the research. There are more Black African men graduating from college today than in prison.

Participant 2: Now I can tell [others]. Do the research (Session 1).

Participant 1, in this excerpt, went on to say he was prompted to visit the library when someone told him that “sixty percent of Black fathers were in prison” and the claim did not ring true to him. In view of this act and the existing literature, we consider how participants might have regarded research and education as forms of critical action to resist oppression (Freire, 1970).

Formerly incarcerated men of color with substance use disorders are stigmatized due to their race, class, gender, involvement with the criminal legal system, and mental health (Alexander, 2010). These experiences are dehumanizing and result in shame, low self‐esteem, and low self‐efficacy (Amaro et al., 2021). Guided by the facilitator, participants critically reflected about their relationships with their communities, often realizing they had more to offer than they had previously believed. For example, one participant relayed, “You can always make positive steps—giving back, helping out as much as you can, giving out knowledge” (Session 3).

However, arriving at realizations like these took some time, and it was not a linear process. Instead, there were windows of awareness that opened and closed throughout the intervention. For instance, in Session 3, one group participant read aloud the statement of purpose from the *Community Wise* handbook. After he read a section describing the community acting together to build empowerment, he stopped and said, “Yeah, that's when they want to shoot you in the head. You getting too powerful” (Session 3). While his remark drew laughter from the rest of the group, we detect a touch of cynicism in this comment, which is an expected outcome of critical reflection (Freire, 1970). Group members were familiar with the tactic of domination that attempts to keep the oppressed ill‐informed and divided against each other, and they knew that organized resistance can be dangerous. Thus, this participant's remark also highlights the potential for detachment that can result if critical reflection produces feelings of inadequacy instead of political efficacy and a willingness to act for change (Goodkind et al., 2020)

Fortunately, over time and with guidance from the peer facilitator, critical consciousness windows opened more frequently as the groups focused on local issues, ways to organize community members, and developing strategies to promote justice and liberation. Analysis of transcripts showed participants were able to understand themselves simultaneously as victims and perpetrators of oppression, but also as agents of change, as described by Freire (1970). Therefore, instead of labelling their commentary as cynicism—when they anticipated backlash for challenging inequities—we recognize they might also have been speaking from an existential perspective that acknowledges a perceived permanence of injustice.

### Radical hope

The participants in *Community Wise* engaged with the radical hope component of the radical healing framework. Reading session transcripts, we made note of perceived growth among participants, who fostered a belief they could repair the damage done/being done by the many faces of oppression and create a better future for themselves and for those in their community. Influenced by multiple orientations (i.e., individual, collective, past, and future), we noticed this sense of radical hope was acted upon and informally practiced by participants throughout the intervention. During the development of his group's Capacity Building Project, one participant stated:

We have to be direct in our approach…because we know there is going to be “noes” and adversaries against it, but we have to continue to have a smiling face, a right attitude—through the good and the bad—because I believe that if we push it, then it could happen (Session 13).

Participants' expressions of radical hope in the intervention were not baseless. The stories of others, like Calvin from the 1990s McDonald's commercials and President Barack Obama, who was the United States' first African American president, motivated participants to imagine possibilities for the individual and collective present and future (Boddie et al., 2024). Yet, not only did we identify discussions with respect to these fictional and non‐fictional people as stimuli for radical hope among participants, but we also acknowledged participants embracing pride in the history of oppression and of their ancestors' resistance to it (Upadhyayula et al., 2017). Emphasizing the knowledge of who you are and where you came from in his definition of family, one participant shared:

With all the technology they got today, they still couldn't build the pyramids…And if we can do that, why can't greater minds do greater things?… But it might not come in my time, but I'm praying that there comes a time, because I never thought that there'd be a Black president (Session 11).

This hope for the future, given a foundation from the past, was a common sentiment voiced by participants. The articulation of the ideas associated with this notion led to rich discussions, including conversations concerning legacy. Although participants communicated an uncertainty in whether the struggles they had experienced had yielded results, they also conveyed a belief they could change things now for greater profit in the future (e.g., to pursue work according to meaning and purpose or to attain something they could pass down to their children).

Participants imagined new possibilities for the future. Finding enjoyment in learning what he can do for his community, one participant's vision included getting his record expunged, getting a loan to fix up a two‐ or three‐family home, and starting “an after‐school program, free of charge,” for young men with difficulties in mathematics or reading (Session 4). We were captivated not only by how demonstrations of radical hope such as these were seemingly accepted by other group members without reservations, but also by how these expressions of radical hope appeared to exceed what some participants thought was possible and allowed others to go beyond their understandings of their possible selves and of their communities (Johnson et al., 2020).

### Strength and resistance

*Community Wise* group participants were actively involved in overcoming adversity. Yet, we witnessed how they were able to maintain faith in/concern for the wellness of the collective and to resist in the face of oppression amid their own healing. Simultaneously, proceeding from their knowledge of the realities of prison and the freedom they enjoyed upon reentry, participants exhibited strength as they experienced hardships and were able to withstand them as they healed. For instance, participants' feelings towards the deprivation of their freedom during incarceration led to unique healing practices. In one group, members bonded over the joy each of them received from engaging in activities such as going to the kitchen and turning the stove on because they did not see fire while they were incarcerated. Other examples of activities included running a bath for themselves due to always having to take showers in prison, as well as randomly walking in and out of rooms, considering they no longer needed to ask for permission. Additionally, one group member exclaimed, “You know how good it feel going to the refrigerator? Opening it?. . I might not even be hungry” (Session 3).

As a result of feelings like these presumably contributing to participants' recognition of oppression, across all groups, discussions materialized related to decision‐making at large and to deciding on what to do with specific feelings (e.g., anger and/or suicidal ideation). From their narratives, we concluded participants believed they could not return to the previous lifestyles that led to their incarceration. Although they differed in their ideas concerning how to resist, overall, their belief resulted in additional acts of resistance. For one participant resistance was “just spending an hour doing something positive” (Session 1). Another participant described how resistance may also be, “if we do like the lobbyist do, get together to push for something; if we all in the community get together, get places where we can come together and talk and come together as a people, one collective mind” (Session 4). Other examples participants gave of to resist oppression included rallying/organizing, education/doing research and taking action, and inspiring others in similar situations. Some participants even advocated for becoming more active in their own legal proceedings, with one participant outlining:

The reason I'm not working is I'm not gonna settle for a minimum wage job. I know that I got a violent criminal past, so only way that I'm gonna get the money I'm capable of making what I deserve is getting my jacket expunged. I'm working with this disability until I can get my jacket expunged, but this ain't enough to live off of, especially when you got a kid (Session 4).

Individual shows of strength and resistance for a common good were acknowledged in our analysis. We recognized several participants developing alternative narratives for themselves and their families. By using his feelings about the system as a motivating force, this participant is creating social change for himself and others. Beyond the ways Jacob et al. (2023) found Black people have utilized to cope with racism (e.g., social support, religion, avoidance, and problem‐focused coping), *Community Wise* provided evidence of collective resistance and reflected People of Color's “commitment to living joy‐filled lives” (French et al., 2020, p. 27). Although participants' accounts did not always consist of healthy responses to oppression (e.g., engaging in behavior deemed criminal, such as assault or substance use), we caught glimpses of participants remaining steadfast in their pursuit of a personal life of joy along with healing for their community. Evidence of their psychological strength was their imagining/organizing rallies for community issues and educating themselves to take the proper action. In addition, transcripts detailed participants' consideration of actions at the individual level, which appeared simple but which we interpret as sophisticated acts of resistance to the dehumanization they had experienced (Rosales & Langhout, 2020). We gather from participants' own words that their actions were aimed at restoring their dignity—fueling the healing process. In the words of the group's facilitator, “This is going to continue. This ain't stopping after the group is over. We're going to pursue this until the end” (Session 14).

### Cultural authenticity and self‐knowledge

The cultural authenticity and self‐knowledge component of radical healing was webbed within each session and, at times, became intermingled with other components of the framework. For example, the question was posed as to why crack cocaine is prosecuted as a “moral issue” versus a “health issue” amongst pregnant women—even though studies indicate alcohol and tobacco use are more prominent and more harmful (Zerai & Banks, 2002). Similar to the dialectic in which radical healing sits, the conversation that followed exemplified an experience cultivated throughout the intervention, especially as it relates to discrimination in BHDC. One participant drew unanimous agreement from the group when he shared his rationale for the inconsistency in the prosecution of crack cocaine:

“cause the government ain't making no money off of it, the government makes money off alcohol and tobacco…It's all about the money man…if they can't control it they not gon” legalize it (Session 2).

Interpreting the participant's response, we consider him having a rationale for the disparity in how crack possession is prosecuted in BHDC to be a demonstration of self‐knowledge (Mosley et al., 2021). The participant challenged the moral assumptions spread by society‐at‐large of BHDC by sharing an alternative theory about his understanding of the government's involvement in the destruction of his community, and the group harmonized in appreciation of his analysis. After identifying similar collective responses to community concerns in other session transcripts, we deduced the men established group cultures that contradicted the assumed predisposition of BHDC to significant social and health difficulties (e.g., drug trafficking and substance use disorders). Fundamentally, we determined that this group—like the others—witnessed a controversial exchange that addressed the internalization of BHDC as morally deficient. Altogether, session transcripts describe how these exchanges were met by group actions that confirmed a collective desire to reject widely distributed information suggesting that People of Color are inferior.

Additionally, the transcript data supports the proposition that sessions served as a setting where members could show up authentically and participate in dialogue addressing racialized experiences without having to code switch. For example, one participant recalled,

Some older White guy…I'm coming out of the store he say “Get out of the way, n‐‐‐‐‐.” Now it was me for being ignorant letting what he said alter my mood. I coulda just let it go over my head, but my ancestors told me to react… so I react and smack him upside his head. Not knowing the police was five minutes away…I was upset with myself because somebody can say what they want to say as long as they don't touch you (Session 1).

This participant spoke freely with his group members about a negative encounter he experienced in the community. Whereas in other spaces his transparency might have been interpreted as indecent, the *Community Wise* group session transcripts chronicled participants engaging in open dialogue with others constantly and reclaiming their identities and the collectivist orientation characteristic of BHDC. Accordingly, we ascertain that the *Community Wise* groups' cultural authenticity reflected a safe space where participants could convey explicit content in addition to displaying passion, remorse, and other feelings that reveal the complexity of emotions associated with experiencing racialized discrimination (Lea et al., 2020). This component of the framework of radical healing accentuates the necessity for People of Color to holistically define themselves without juxtaposition to Whiteness and/or colonization (French et al., 2020). In addition to a focus on living in one's truth as an active form of resistance and despite oppressive forces, the cultural authenticity and self‐knowledge component emphasizes celebration and outward displays of racial and ethnic behaviors which the participants in *Community Wise* exhibited.

### Collectivism or emotional and social support

*Community Wise* group sessions included activities that helped participants establish group norms and peer social emotional support (Yalom & Leszcz, 2020). The optimized groups were conducted by a peer facilitator. He was from the same community and had the same racial‐ethnic background as most of the participants, in conjunction with sharing similar histories of incarceration and substance use. From our analysis of the group sessions, the peer facilitator's contribution to the group appears to have extended beyond simply implementing the curriculum. For instance, sessions started with a check‐in where the facilitator asked each group member to share how he was feeling that day. In the first group session, the peer facilitator was vulnerable and transparent about his anguish and resolve that stemmed from his own history with incarceration and substance use. Based on participants' responses to his self‐disclosure in session, we identify the facilitator's transparency as an aspect that cultivated a welcoming environment and set the foundation for meaningful dialogue where participants could unpack decades of internalized and external oppression (Yalom & Leszcz, 2020).

Ultimately, the check‐ins encouraged group members to share their emotions/feelings, and we witnessed how participants were unwavering in their desire to preserve established group norms. For example, during one session, when a participant was late, his fellow group member ensured the facilitator completed his check‐in.

Ask [participant] how was his weekend, man…he might have something on his mind he need to get off, man… then I'm going to feel bad… if he go out there and kill someone or something, I could've talked to him (Session 7).

This statement is an example of many instances where group members showed a willingness to hold space for each other. In this example, the participant who was late revealed his nephew was waiting for a heart transplant, which broadened the group's dialogue on faith and death. His being vulnerable by sharing his family's disposition encouraged other group members to do the same, and later—in the same session—a few other participants disclosed their experiences with death and with loved ones having terminal illnesses. Throughout the *Community Wise* sessions and especially in instances where participants shared their personal narratives, such as this, we noticed participants build solidarity—which the radical healing component of collectivism or emotional and social support demands. In Session 3 of his group, one participant identified threats in his community (e.g., corruption, disease, (internalized) oppression, peer pressure, poverty, and unemployment) in addition to resources he could use to combat them (e.g., social support)—professing “this is my social support; being here.” Transcripts revealed how participants not only held space for one another but also affirmed the various responses to a whole host of topics, including illness, death, and other losses. Similarly, we observed how the groups contributed to a sense of collectivism, particularly when working on capacity‐building projects (Gomez et al., 2024). When reflecting critically on structural oppression of BHDC in the CBP sessions, we beheld participants speaking as members of those communities developing collective capacity to act in resistance to oppression.

## IMPLICATIONS

Exploring the *Community Wise* intervention through a radical healing lens illuminated several instances in which the components of radical healing emerged while participants discussed their experiences with oppression and resistance. Therefore, our analysis provides evidence that the dimensions of radical healing (i.e., critical consciousness; radical hope; strength and resistance; cultural authenticity and self‐knowledge; and emotional or social support) were present in *Community Wise* participants' personal narratives. The intervention's critical consciousness foundation provided opportunities for participants to practice critical reflection and evaluate and/or modify their plans for critical action. Analysis of session transcripts revealed how engaging participants in critical reflection on inequities and developing plans for collective action encouraged the groups to organize around community‐level issues and address structural racism. This finding, in particular, suggests that the *Community Wise* intervention extended beyond individual treatment and promoted a multisystemic approach to wellness that considers the multiple interconnected systems of oppression. Although measuring sustained community engagement was beyond the scope of the optimization study, future research should explore whether an intervention grounded in the psychological framework of radical healing and organized around identification with communities and collective resistance might enable participants to create structures that support positive treatment outcomes (e.g., mental health and psychological well‐being) long‐term.

Linking our findings back to the radical healing literature, this study answers scholars' calls for future research that shifts away from traditional, individual ideas of healing, focuses on the strengths of communities of color, and utilizes a plethora of research methods (French et al., 2020). Responding to these requests, our study of the *Community Wise* intervention investigated a radical, collective notion of healing, centered the voices of the People of Color, and employed a qualitative methodology. Apart from providing rich narratives of the complex process, generally, this study offers specific examples of radical healing among people who are often underrepresented in research (i.e., People of Color with incarceration and/or substance use histories). Consequently, we believe we have contributed new perspectives to what is known about radical healing and laid a foundation for future inquiry that incorporates variables beyond the individual. In the future, it is recommended that researchers consider more structural variables when exploring radical healing to expand descriptions of ways that communities of color can heal from racism and oppression.

French et al. (2020) also highlight the healing and transformative power of the testimony—which emerged as a unique pattern/practice stemming from the safety of the *Community Wise* groups. For example, some *Community Wise* participants offered personal narratives of their struggles with substance use and reactions to intense emotions, while others expressed being pessimistic when considering their lack of resources and individual change. Concurrently, participants communicated a unified dissatisfaction with the divided state of their community and charged other community members to contemplate their contributions to the current condition. Though not all participants were able to identify wounds and/or culturally‐informed healing practices, for those who did, personal narratives and individual accounts should be properly understood as naming discrimination and their racialized experiences. Throughout the groups, they gave a voice to their oppression, and their experiences were validated. Moving forward, clinicians are encouraged to create innovative community programs that provide more opportunities for healing practices—with an emphasis on group and communal witness bearing—so others can be empowered toward meaning making and envisioning future possibilities.

## LIMITATIONS

One issue for consideration is the inclusion of the original developers of *Community Wise* as co‐authors. Although their perspectives offer valuable background knowledge of and experience with the intervention, their involvement may have influenced how the larger research team interpreted the data. Consequently, co‐authors who were new to the work were included, and reflexivity was encouraged to challenge the team's thinking and assumptions concerning the data. Ultimately, we believe having the original developers of the intervention serve as co‐authors enhanced our understanding and the depth of our interpretations.

Another issue for consideration is that the original study did not employ the radical healing framework in its development. This fact resulted in radical healing concepts beyond critical consciousness not being incorporated in the design of the intervention. Radical healing was, therefore, applied after the fact. Accordingly, there is a possibility that the interpretation of the findings may have been influenced by confirmation bias.

Despite these considerations, this study offers novel insights into how elements of radical healing can emerge organically even when not incorporated into the design of an intervention. In this study, participants were guided and actively worked towards critical consciousness, specifically. However, while not embedded into the intervention by design, the other components of the psychological framework of radical healing were found in participants' rich narratives. Participants' narratives revealed an iterative process of radical healing—evidenced by all five of the framework's components being identified in participants' own words.

## CONCLUSION

Overlaying the radical healing framework onto *Community Wise* suggests that engagement in CD and CBP helped participants start healing from internalized oppression and confront systemic racism, while simultaneously reconciling their location in relation to their various social identities (i.e., positionality). Sharing testimony in groups of individuals with histories of oppression in common, guided by a peer facilitator and an empowering curriculum, helped participants navigate an iterative process of recognizing systems of discrimination and envisioning liberation through community solidarity. Incorporating the radical healing framework when developing innovative and community‐engaged multisystemic group interventions, such as *Community Wise*, may yield improved recovery outcomes beyond the limits of traditional approaches. This expanded focus incorporates personal healing and fosters a collective sense of empowerment and resilience within the community.

## CONFLICT OF INTEREST STATEMENT

The authors declare no conflicts of interest.
